# Development of Novel Composite Core Using Powdered Macadamia Nutshell and Its Sandwich Structures for Building and Other Engineering Applications

**DOI:** 10.3390/ma18184369

**Published:** 2025-09-18

**Authors:** Md Mainul Islam, Sutirtha Chowdhury, Md Sefat Khan

**Affiliations:** 1 Centre for Future Materials, Institute for Advanced Engineering and Space Sciences, University of Southern Queensland, Toowoomba, QLD 4350, Australia; sutirtha.chowdhury@unisq.edu.au (S.C.); mdsefat.khan@unisq.edu.au (M.S.K.); 2 School of Engineering, University of Southern Queensland, Toowoomba, QLD 4350, Australia

**Keywords:** green composites, macadamia nutshell, bio-based materials, sandwich structures, sustainable construction

## Abstract

Growing environmental concerns and the depletion of fossil-based resources have accelerated the demand for sustainable alternatives in engineering and construction materials. Among these, bio-based composites have gained attention for their use of renewable and eco-friendly resources. Macadamia nutshells, typically treated as agricultural waste, possess high strength, brittleness, heat resistance, and fracture toughness, making them attractive candidates for structural applications. Australia alone contributes nearly 40% of global macadamia production, generating significant shell by-products that could be repurposed into high-value composites. This study investigates the development of novel composite cores and sandwich structures using macadamia nutshell particles reinforced in an epoxy polymer matrix. Two weight ratios (10% and 15%) and two particle sizes (200–600 µm and 1–1.18 mm) were employed, combined with laminating epoxy resin and hardener to fabricate composite cores. These cores were further processed into sandwich specimens with carbon fabric skins. Flexural and short beam shear (SBS) tests were conducted to evaluate the mechanical behaviour of the composites. The results demonstrate that higher filler content with fine particles achieved up to 15% higher flexural strength and 18% higher stiffness compared to coarser particle composites. Sandwich structures exhibited markedly improved interlaminar shear strength (8–15 MPa), confirming superior load transfer and durability. The results demonstrate that higher filler content and finer particles provided the most favourable mechanical performance, showing higher flexural strength, stiffness, and shear resistance compared to coarser particle formulations. Sandwich structures significantly outperformed core-only composites due to improved load transfer and resistance to bending and shear stresses, with the 15% fine-particle configuration emerging as the optimal formulation. By transforming macadamia nutshells into value-added composites, this research highlights an innovative pathway for waste utilisation, reduced environmental impact, and sustainable material development. The findings suggest that such composites hold strong potential for structural applications in construction and related engineering fields, especially in regions with abundant macadamia production. This study reinforces the role of agricultural by-products as practical solutions for advancing green composites and contributing to circular economy principles.

## 1. Introduction

Rising apprehensions over environmental deterioration and the progressive exhaustion of fossil fuel reserves have amplified worldwide initiatives to create sustainable substitutes for traditional materials. The transition to bio-based materials has garnered considerable momentum, not only for its ecological advantages but also as a feasible substitute for conventional fossil-derived materials. Principles of the circular economy are fundamentally anchored in concepts such as life cycle analysis, sustainability, green chemistry, and eco-friendly product design, aiming to establish regenerative systems that harmonise economic, environmental, and societal requirements. This concept promotes a closed-loop system of production and consumption designed to improve resource efficiency and reduce environmental impact [1]. A composite material generally comprises two separate components: a matrix phase and a reinforcement, integrated to produce improved functional qualities. Recently, the advancement of green composites, consisting of biodegradable matrices and natural reinforcements, has surfaced as a viable remedy to the constraints of petroleum-based composites. These advanced materials provide enhanced functionality while minimising environmental impact. Recent studies have highlighted the significant potential of incorporating agricultural by-products such as macadamia nutshells into structural composites, demonstrating improvements in mechanical and thermal properties while contributing to sustainable waste utilisation [2,3,4,5]. Moreover, the environmental behaviour of macadamia-PLA biocomposites, including biodegradation and water absorption characteristics, has also been elucidated in recent research, offering valuable insights into their sustainability and long-term performance [6]. As awareness of limited petrochemical reserves and the necessity to diminish carbon footprints increases, green composites are being increasingly investigated across several industries [7]. Originating from renewable sources, these materials can be safely discarded or composted at the end of their lifecycle, thereby favourably impacting the natural carbon cycle. Nonetheless, despite their ecological benefits, green composites have not yet attained the performance reliability and scalability necessary for extensive industrial implementation. Additional research is required to enhance the procurement and processing of their components to guarantee long-term sustainability. Historically, advanced composites have relied on high-strength synthetic fibres, such as carbon, Kevlar, and glass, integrated with thermoset resins, including epoxies and poly(etheretherketone) (PEEK). These materials are extensively utilised in aeronautical, automotive, and civil engineering applications because of their superior strength-to-weight ratios and moldability. However, they possess significant disadvantages: their basic materials originate from non-renewable petroleum resources, which are swiftly diminishing, with forecasts predicting depletion over the next 50 to 60 years. Moreover, thermoset-based composites are famously challenging to recycle, frequently concluding their lifecycle in landfills where they last for millennia. Increasing regulatory pressure and public apprehension regarding trash accumulation have expedited the shift towards sustainable alternatives [8].

Researchers have sought biopolymers and natural fibres as alternatives to synthetic matrices and reinforcements. These advances have resulted in significant advancements in sustainable materials science, with natural fibre-reinforced composites currently utilised in industries such as construction, automobile production, and packaging [9,10,11]. In addition to natural fibres, there is a growing focus on agricultural byproducts, materials that are presently underutilised or abandoned. Macadamia nutshells have demonstrated notable potential. Khan et al. [4] suggested that macadamia shells are frequently incinerated as biomass, utilised in organic fertilisers, or discarded. These shells have distinctive structural and mechanical characteristics, including high strength, low density, and an isotropic cellular structure, rendering them appealing options for composite applications. Indigenous to Australia, macadamia nuts are associated with an industry that produces over 28,000 tonnes of shell trash each year, as global demand persists in increasing. Considering that shells comprise around 70% of the overall fruit weight, there exists considerable opportunity for recycling this plentiful residue. Macadamia nutshells have developed remarkable toughness and mechanical resistance in their structure. Their cellular structure integrates lightweight properties with durability, presenting advantageous applications in sandwich composites, structures recognised for their superior strength-to-weight ratios and energy absorption potential. Macadamia nutshell fillers, when integrated into polymer matrices, may facilitate the development of novel, eco-friendly components for infrastructure, aerospace, and automotive applications [4]. This research project seeks to examine the production methods and fundamental characteristics of a composite core created from powdered macadamia nutshells, emphasising its relevance in engineering applications. Ongoing enhancements continue to be recognised in the composite materials and their applications. One study [2] investigated the potential of various agricultural wastes, including macadamia nutshells, to generate outstanding composites. These investigations underscore the relevance of composite materials in developing ecological engineering solutions and mitigating environmental problems [3].

## 2. Macadamia Nutshell and Prior Research

**Figure 1:**
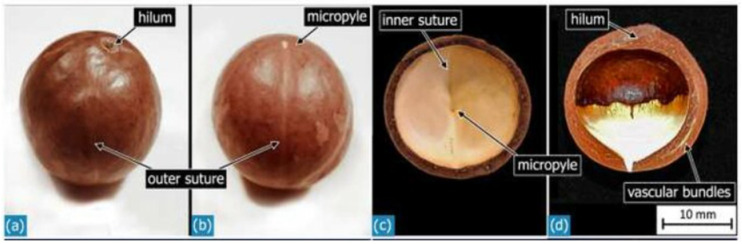
Components of the macadamia nutshell with its structure: (**a**,**b**) outer surface; (**c**,**d**) inner surface [4].

Macadamia nutshells, categorised as agricultural waste, consist of lignocellulosic materials. Macadamia nutshell, a lignocellulosic residue typically comprising ~47.6% lignin, 25.8% cellulose, and 11.7% hemicellulose [12,13], serves as a low-cost, low-density filler that imparts partial biodegradability to the resulting materials [14,15,16]. The primary source of strength and stiffness in these agro-fibres is their cellulose content. Historically, nutshells have been relegated to low-end applications; however, recent investigations have begun to explore more valuable and innovative uses for them. Current investigations are focusing on the use of nutshells as carriers for insecticides and pesticides, as well as their potential as abrasives in cleaning and polishing products, and as raw materials to produce activated carbon. Furthermore, recent studies have thoroughly investigated the potential of utilising nutshells as filler materials in polymer composites [4]. The shells and other by-products, which make up 70% of the nut’s weight, are employed in a variety of applications. Additionally, these shells serve as a biomass fuel source with a calorific value of 5500 kcal/kg and are utilised as a filler material in the plastic industry [10]. Wechsler et al. [10] showed that Macadamia nutshells consist of cellulosic fibres, which have a distinctive structure that is isotropic, with fibres arranged randomly, unlike the organised fibres found in wood. The modulus of elasticity for the nutshell is noted to range from 4.2 to 5.2 GPa. Similarly, they exhibit a tensile strength of 55 MPa. The bulk density is recorded at 680 kg/m^3^, while the total density stands at 1127 kg/m^3^, accompanied by a moisture content of approximately 10%. The thickness of these shells ranges from 1.5 mm to 5 mm, and they consist of two distinct layers: a softer internal layer and a harder external layer. Khan et al. [4] delineated the anatomical components of Macadamia nutshells, which comprise the hilum, micropyle, outer suture, vascular bundles, sclerenchyma fibre layer, sclereid, and test layers (Figure 1). These terms form a botanical lexicon crucial for understanding shells from both material science and mechanical viewpoints.

The advantages and disadvantages of incorporating macadamia nutshells in bio-synthetic polymer composites were analysed by Khan et al. [4]. The incorporation of macadamia nutshell particles into polymer matrices significantly enhances the tensile strength, flexural strength, and impact resistance of the composites studied. The small size, hardness, and strong adhesion characteristics of the nutshell bits to the polymer matrix are the reasons for this phenomenon. Liu et al. [17] examined the incorporation of macadamia nutshell particles in cement-based mortars. The study demonstrated that these particles markedly enhanced the strength of the mortars in both compression and bending. The research demonstrated that agricultural waste can be utilised to create high-performance building materials, providing environmental advantages and minimising waste. Cortat et al. [18] explored macadamia nutshell residue as a sustainable filler in polypropylene by varying the loading from 5–30 wt%, then assessed the composites’ physicochemical, mechanical, morphological, thermal, and water-uptake behaviour; they also performed a life-cycle assessment to evaluate sustainability impacts.

## 3. Experimental Observations

### 3.1. Material Selection

The experimental initiatives began with the creation of composite cores by integrating 10% and 15% weight ratios of macadamia nutshell of two sizes, specifically 200–600 µm and 1–1.18 mm, combined with 240 parts of epoxy resin and 60 parts of laminating hardener. Similarly, sandwich structures were created by layering carbon fabric as a thin outer layer on the composite cores. Furthermore, mechanical tests were performed to examine the properties and characteristics of these composites.

### 3.2. Sample Preparation

In the next phase, the mixture of nutshell particles and epoxy resin and hardener is made. For mixture 1, which is a 10% weight ratio: 240 parts of epoxy resin and 60 parts of hardener is mixed with 30 g of nutshell of both sizes. Similarly, for mixture 2, which is a 15% weight ratio here 240 parts of epoxy resin and 60 parts of hardener are mixed with 45 g of nutshell. The epoxy resin to hardener mixing ratio (240:60 by weight) was strictly based on manufacturer recommendations to ensure optimal curing and mechanical performance. This quantity is used for both sizes to get two sizes of composite panels with the same quantity of materials. The epoxy resin, hardener, and nutshells are all measured using a weighing machine the subsequent process, the macadamia nutshells are assessed for enough dryness; if insufficient, they are positioned on an oven tray and subjected to the oven for 5–10 min at 70–80 °C for pre-drying. In the subsequent phase, after thoroughly mixing the macadamia nutshell, resin, and hardener for approximately 8–10 min, the slurry for each size, according to varying weight ratios, is poured into the moulds according to their designated markings. For 10% with a size of 200–600 µm in a different mould, and for 10% with a size of 1–1.18 mm in a separate mould. Similarly, the 15% slurry with a particle size of 200–600 µm is positioned in distinct moulds, while the 15% slurry with a particle size of 1–1.18 mm is placed in separate moulds. The moulds are maintained at room temperature for approximately 24 h for curing. The moulds undergo post-curing in the oven for approximately 8 h at 80 °C.

**Figure 2:**
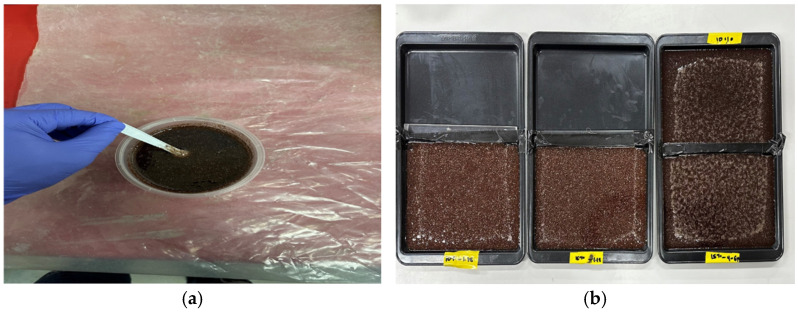
(**a**) Making of the slurry (mixing: resin, hardener, and nutshell); (**b**) moulds with slurry (kept at room temperature for curing).

In Figure 2, the mixing of slurry is shown with resin, hardener and shell; after this stage, moulds were made from the slurry and were kept at room temperature for curing. Upon completion of post-curing, the composite panels were carefully extracted from the moulds, cleaned, and subsequently bisected. One-half was further subdivided into smaller dimensions according to the testing criteria, while the other half was reserved for sandwich manufacturing.

**Figure 3:**
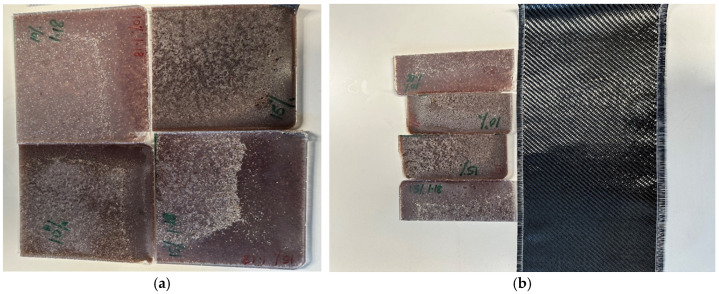
(**a**) Composite core removed from mould; (**b**) half-cut core for sandwich fabrication visualisation.

**Figure 4:**
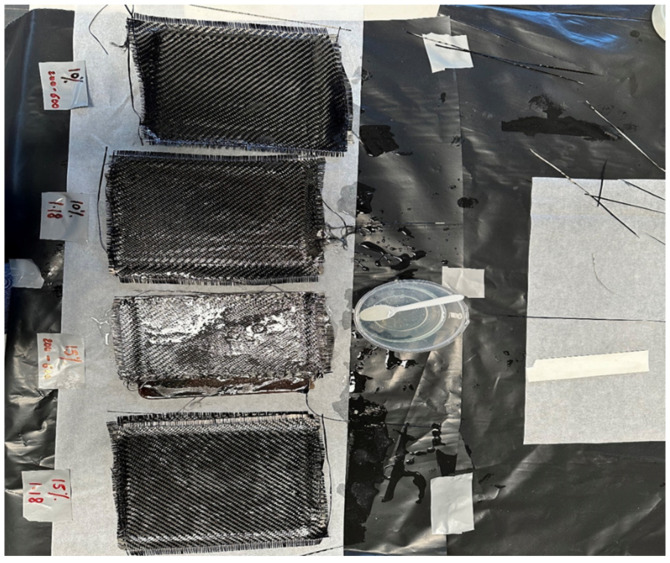
Fabrication of sandwich structure of composite.

Figure 3 shows the core composites which were removed from the mould and cut in half for fabrication. In the following phase, the sandwich structures were formulated. Figure 4 shows the fabrication of the sandwich structure of the composite. Initially, 240 parts of epoxy resin and 60 parts of hardener by weight were combined in a container and thoroughly mixed. Subsequently, carbon fabric skins were tailored to the dimensions of the core. The carbon fabric used as skin was a plain weave with an areal density of 200 g/m^2^, cut to the dimensions of the core specimens. This specification strongly influences interlaminar shear strength. Two carbon fabric skins were cut for each core. The matrix mixture was applied to the core panels using a brush, and skins were positioned on the top and bottom of the core panel, followed by an additional layer of matrix on the top and bottom skins. The material was subsequently cured for an additional day, followed by post-curing in an oven for two hours at 60 degrees Celsius.

Flexural and short-beam shear (SBS) tests were chosen to be performed on the prepared specimens as they directly represent the load-bearing and interlaminar behaviour relevant to structural applications such as sandwich panels and construction materials. Each test was repeated three times to ensure repeatability, and the mean values were reported with observed variations within ±5%.

## 4. Results and Analysis of Flexural Test

Flexural testing is an important way to determine the mechanical properties of composite materials by testing how well they can bend under a transverse force. According to ASTM D790 standards [19], the three-point bending test was done on both core-only composites and sandwich panel specimens. Dong & Davies (2012) evaluated the flexural behaviour of polyester composites reinforced with macadamia nutshell particles using a three-point bending test, in which a loading nose deflects the specimen over a fixed span at a set rate until failure. In this study, the main goal was to find out how different amounts of filler (10% and 15% by weight) and particle sizes (200–600 µm and 1–1.18 mm) affected the load-bearing capacity, deflection behaviour, and failure characteristics. The load–deflection curves showed that the flexural strength and modulus were greatly affected by both the amount of filler and the size of the particles. Specimens with 15% filler and smaller particles (200–600 µm) consistently performed better than other configurations, showing higher peak loads and more deflection before failure. On the other hand, panels with 10% filler and coarser particles (1–1.18 mm) showed the worst flexural performance, with brittle fractures and less energy absorption.

Most of the time, failure started on the tensile side of the specimen and moved up. Samples with finer fillers showed more cohesive fracture surfaces, while composites with coarse particles showed more irregular and porous failure regions.

### 4.1. Summary of Flexural Test Results

There were clear differences in the load-deflection curves for all the specimens based on the composite formulation:

Core-only composites with 15% filler (200–600 µm) could hold the most weight without breaking, and when they did, it was smooth and gradual, which showed that the matrix and filler were interacting well and absorbing energy. On the other hand, Core-only composites with 10% filler (1–1.18 mm) failed early, had lower peak loads, and broke in a brittle way. This suggests that stress transfer was poor because the particles were not evenly spread out and the bonding was not strong. The failure usually started on the tensile side in the middle of the span and moved up. Finer fillers helped cracks spread evenly, while coarse fillers made areas of stress concentration that sped up the fracture.

The carbon fibre skins made the sandwich panels bend better in this case. The sandwich panels with 15% filler (200–600 µm) were the most flexible. They could hold more weight and bend a lot before breaking. Panels with 10% filler (1–1.18 mm) broke too soon because the core cracked or delaminated, but the skin didn’t change shape much. The flexural strength values (based on three-point bending tests) for each composite group are summarised in Table 1 below:

Increasing the filler proportion from 10% to 15% improved flexural performance across all particle sizes, particularly with finer particles. Sandwich panels outperformed core-only composites due to the effectiveness of carbon fibre skins in withstanding tensile and compressive stresses. Reduced particle sizes (200–600 µm) enhanced mechanical interlocking and stress distribution within the matrix, hence increasing overall durability.

In Table 2 below, the flexural calculations for all specimens are listed:

### 4.2. Summary of Short Beam Test Results

The short beam shear (SBS) test was utilised to evaluate the interlaminar shear strength (ILSS) and the structural integrity of carbon fibre-reinforced sandwich composites containing macadamia nutshell-filled epoxy cores. This test provides essential insight into the performance of the core-skin interface, frequently the initial point of failure in bending and out-of-plane loading scenarios. The findings are analysed concerning variations in filler content (10% and 15%) and filler particle size (200–600 µm and 1–1.18 mm). ILSS values obtained in this study are comparable to those of other bio-based particulate composites reported in the literature, typically in the range of 8–15 MPa. Our results fall within this range, demonstrating competitive performance.

Under the SBS test, the composite structure experiences maximum shear stress at the mid-plane between the loading nose and the support points. In sandwich panels, this stress is distributed across (a) the skin–core interface, where delamination may occur if adhesion is poor, (b) the core itself, which must resist out-of-plane shear and (c) the resin–filler matrix, which transfers load across the thickness. In the SBS test 15% filler panels worked better because they were stiffer and held together better inside. The higher filler content made the core’s effective modulus stronger and made it less likely to crack, especially in composites with fine fillers. 10% of filler panels had a lower shear load capacity, especially those with bigger particles. The lower filler concentration made the matrix less continuous and less able to resist core shearing.

**Figure 5:**
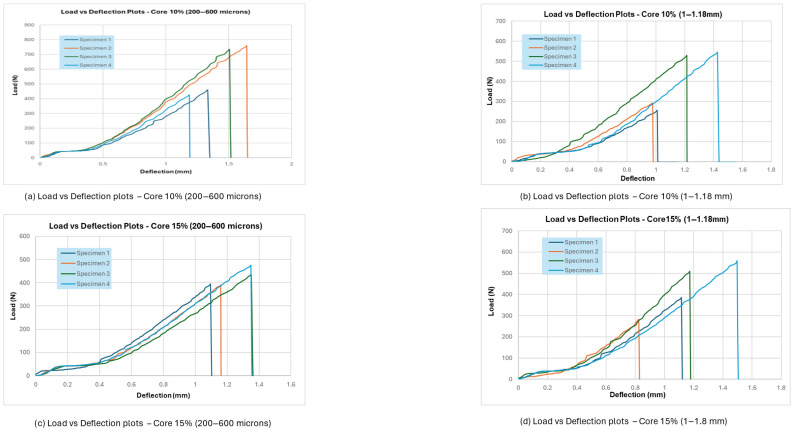
SBS Test result—for core specimen.

**Figure 6:**
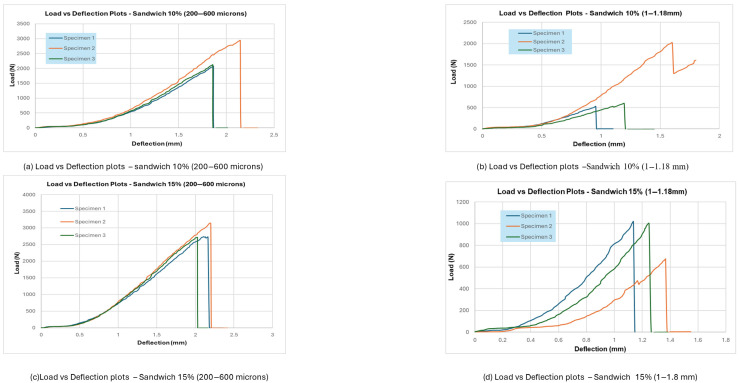
SBS test result—for sandwich specimen.

From Figure 5 and Figure 6, it can be observed that larger particles make composite materials less uniform and less ductile because they create weak points in the matrix. The higher concentration of macadamia nutshells (15%) also plays a role in the different mechanical responses seen between the two particle size distributions. In general, smaller particles perform better when it comes to taking on loads and deforming. Moreover, finer particles (200–600 µm) allowed for even stress transfer and better mechanical interlocking with the epoxy matrix. This made the core more resistant to shear and delayed delamination. Larger particles (1–1.18 mm) acted as stress concentrators, which started cracks at the interface between the particle and the resin. This caused early failure modes like debonding and core splitting when the shear loads were low. Below, Table 3 represents the observations from the SBS test.

## 5. Additional Characterisation

### 5.1. FTIR on Macadamia Nutshell

**Figure 7:**
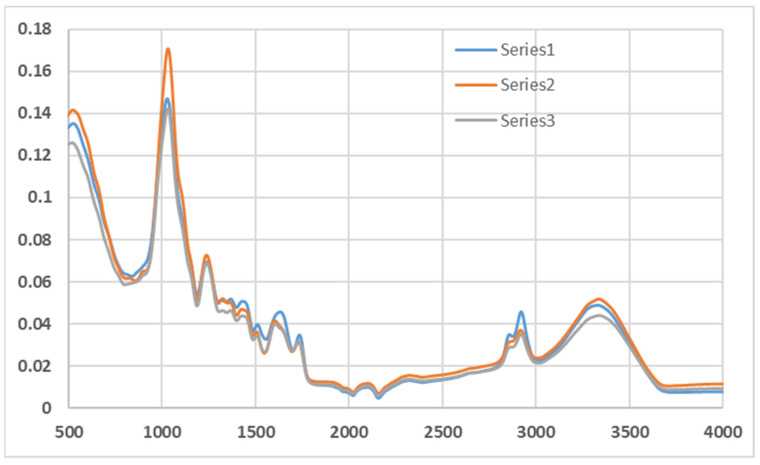
FTIR spectrum (Transmittance vs. Wavenumber in cm^−1^) for macadamia nutshell powder.

Fourier Transform Infrared (FTIR) spectroscopy was carried out on macadamia nutshell powder to examine the functional groups present. The FTIR spectrum (Figure 7) revealed characteristic peaks corresponding to lignocellulosic components, including O–H stretching (~3330 cm^−1^), C–H stretching (~2915 cm^−1^), C=O stretching (~1730 cm^−1^), and aromatic skeletal vibrations (~1600 cm^−1^). These results confirm the presence of cellulose, hemicellulose, and lignin, which contribute to the filler’s chemical reactivity.

### 5.2. TGA on Composites

**Figure 8:**
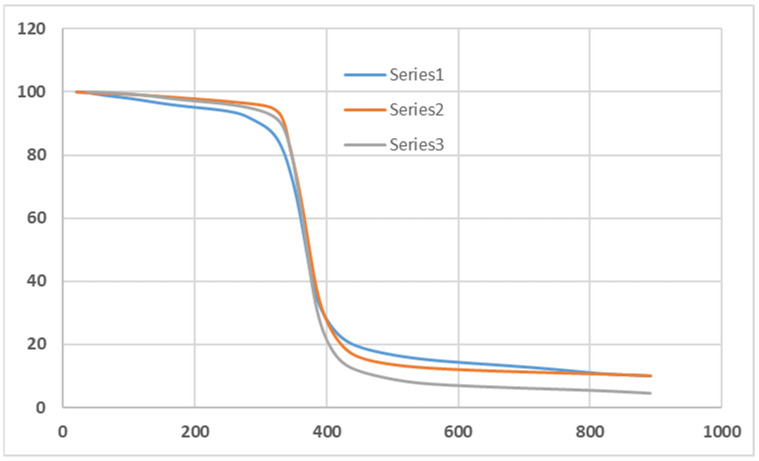
TGA curves (weight % retained vs. temperature in °C) for 15 wt% macadamia nutshell epoxy composites.

Thermogravimetric analysis (TGA) was performed on composites containing 15 wt% macadamia nutshell in epoxy resin (Figure 8). The TGA results demonstrated a three-step degradation process. Initial weight loss below 120 °C corresponded to moisture removal, followed by hemicellulose and cellulose degradation (200–350 °C), and lignin degradation extending up to 500 °C. The char residue observed above 500 °C indicates enhanced thermal stability of nutshell-filled epoxy composites compared to neat resin.

## 6. Conclusions and Recommendations

### 6.1. Conclusions

This research primarily examined the qualities, features, and mechanical behaviour of composite materials composed of macadamia nutshell particles reinforced with a polymer matrix. This investigation included two weight changes: 10% and 15%, alongside two particle size variations of 200–600 µm and 1–1.18 mm. Two weight variations (10% and 15%) and two nutshell size variations (200–600 µm and 1–1.18 mm) were combined with 240 parts of Thixotropic laminating epoxy resin (Kinetix R246TX, from ALT Composites, QLD, Australia) and 60 parts of laminating hardener (H160, from ALT Composites, QLD, Australia) to fabricate composite core specimens, onto which carbon fabric was applied as a skin to create sandwich specimens. This research primarily comprises two assessments: the Flexural Test and the Short Beam Test conducted on the prepared specimens according to the testing standards. The principal conclusions derived from this study endeavour are summarised as follows:

I Given that macadamia nutshells exhibit a robust structure and contain mechanical features such as brittleness, considerable strength, heat resistance, and high fracture toughness, coupled with Australia accounting for 40% of global output, their utilisation should be prioritised in the manufacture of composite materials.
II A composite core with a 10% weight ratio and a size range of 200–600 µm demonstrates superior flexural strength, increased deflection, enhanced flexural and short beam loads, and more short beam strength when compared to a composite core with a 10% weight ratio and a size range of 1–1.18 mm.
III A composite core with a 15% weight ratio and a size of 1–1.18 mm exhibits superior values of flexural stress, flexural strain, and short beam shear strength in comparison to a composite core with a 15% weight ratio and a size of 200–600 µm.
IV Sandwich specimens with a 10% weight ratio and a size of 200–600 µm exhibit enhanced flexural stress, flexural strain, improved flexural and short beam deflection, and superior shear strength compared to sandwich specimens with a 10% weight ratio and a size of 1–1.18 mm.
V The sandwich with a 15% weight ratio and a size of 200–600 µm demonstrates superior flexural stress, flexural and short beam loads, and short beam strength compared to the sandwich with a 15% weight ratio and a size of 1–1.18 mm.
VI According to the flexural test data, the 15% sandwich with a particle size of 200–600 µm exhibits the highest load and stress, rendering it acceptable for high-load applications, whereas the 10% core with a size of 1–1.18 mm demonstrates the lowest load value, making it unsuitable for such applications.
VII According to the flexural data, a 15% sandwich with a size of 1–1.18 mm exhibits the maximum deflection, rendering it more flexible and resilient under stress, whereas a 15% core with a size of 200–600 µm has the lowest deflection, resulting in increased stiffness and reduced flexibility.
VIII The short beam test data indicates that the sandwich with a 15% weight ratio and a particle size range of 200–600 µm exhibits the maximum load and shear strength values, rendering it exceptionally resilient for high-load applications.
IX According to the Short Beam test data, a 10% core with a size of 1–1.18 mm exhibits the lowest load capacity, while a 15% core with a size of 200–600 µm demonstrates the lowest shear strength, rendering it stiffer and unsuitable for high-load applications. Overall, sandwich specimens with a weight ratio of 15% and a size range of 200–600 µm are identified as the optimal weight ratio and size range, respectively, based on the research findings.

### 6.2. Future Recommendations

I Only two weight ratios, 10% and 15%, of macadamia nutshell were employed in this research for the fabrication of composite cores and their sandwich structures; therefore, future studies should incorporate and evaluate additional weight variations, such as 25% and 30%.
II For this research, just two particle size ranges were utilised: 200–600 µm and 1–1.18 mm. Consequently, alternative particle sizes, such as 100–200 µm and 1–1.5 mm of macadamia nutshell, may be employed and evaluated.
III In this research, the hand lay-up approach was utilised to fabricate composite and sandwich specimens; however, alternative procedures such as solution casting and extrusion may be employed in future studies.
IV For this research, carbon fabric was utilised as the outer layer for constructing sandwich specimens; in the future, other thicker, softer, and stronger materials may be employed and evaluated for sandwich applications.
V Environmental durability factors such as moisture uptake and thermal cycling may be employed in future studies.

## Figures and Tables

**Table 1:** Flexural strength value for each component (Three-point bending test).

Type	Filler %	Particle Size (µm)	Max Flexural Load (N)	Failure Mode
Core Only	0.1	200–600	112	Matrix cracking
Core Only	0.1	1–1.18 mm	98	Brittle fracture
Core Only	0.15	200–600	128	Uniform shear fracture
Core Only	0.15	1–1.18 mm	119	Shear + filler debonding
Sandwich Panel	0.1	200–600	138	Localised core crushing
Sandwich Panel	0.1	1–1.18 mm	122	Skin–core debonding
Sandwich Panel	0.15	200–600	165	Progressive matrix cracking
Sandwich Panel	0.15	1–1.18 mm	146	Mixed-mode fracture

**Table 2:** Average flexural calculation of all specimens.

Specimen Type	Nutshell Size	Average Load (N)	Average Maximum Deflection (mm)	Average Flexural Stress (MPa)	Average Flexural Strain (%)	Average MOE (MPa)
10% Core	200–600 µm	158.775	6.689	64.487	2.49	2693
10% Core	1–1.18 mm	124.94	5.051	57.085	1.98	3119.96
15% Core	200–600 µm	139.16	4.859	50.387	1.79	3015.75
15% Core	1–1.18 mm	129.735	5.629	53.105	2.08	2783
10% Sandwich	200–600 µm	619.03	7.206	142.676	2.38	7550.66
10% Sandwich	1–1.18 mm	552.11	6.3174	143.56	2.21	10,772.33
15% Sandwich	200–600 µm	750.43	6.783	162.52	2.093	10,221.67
15% Sandwich	1–1.18 mm	515.45	8.95	119.36	2.65	4639.3

**Table 3:** SBS test observations and failure modes according to specimens’ configurations.

Configuration	Filler %	Particle Size	Max Shear Load (N)	Observed Failure Mode	Analysis Summary
10%—200–600 µm	10%	200–600 µm	102	Interface delamination	Moderate load capacity. Improved bonding vs. coarse fillers, but limited cohesion.
10%—1–1.18 mm	10%	1000–1180 µm	88	Skin–core debonding	Lowest performance. Coarse particles led to weak bonding and early interfacial failure.
15%—200–600 µm	15%	200–600 µm	118	Core shear rupture (cohesive failure)	Highest load bearing. Strong interlocking and improved matrix continuity.
15%—1–1.18 mm	15%	1000–1180 µm	109	Mixed shear + delamination	Acceptable performance. Increased filler improved strength despite coarse texture.

## Data Availability

The original contributions presented in this study are included in the article. Further inquiries can be directed to the corresponding author.
